# Twilight, a Novel Circadian-Regulated Gene, Integrates Phototropism with Nutrient and Redox Homeostasis during Fungal Development

**DOI:** 10.1371/journal.ppat.1004972

**Published:** 2015-06-23

**Authors:** Yi Zhen Deng, Ziwei Qu, Naweed I. Naqvi

**Affiliations:** 1 Temasek Life Sciences Laboratory, Department of Biological Sciences, National University of Singapore, Singapore; 2 Guangdong Province Key Laboratory of Microbial Signals and Disease Control, College of Agronomy, South China Agricultural University, Guangzhou, China; 3 School of Biological Sciences, Nanyang Technological University, Singapore; Chinese Academy of Sciences, CHINA

## Abstract

Phototropic regulation of circadian clock is important for environmental adaptation, organismal growth and differentiation. Light plays a critical role in fungal development and virulence. However, it is unclear what governs the intracellular metabolic response to such dark-light rhythms in fungi. Here, we describe a novel circadian-regulated *Twilight* (*TWL*) function essential for phototropic induction of asexual development and pathogenesis in the rice-blast fungus *Magnaporthe oryzae*. The *TWL* transcript oscillates during circadian cycles and peaks at subjective twilight. GFP-Twl remains acetylated and cytosolic in the dark, whereas light-induced phosphorylation (by the carbon sensor Snf1 kinase) drives it into the nucleus. The mRNA level of the transcription/repair factor *TFB5*, was significantly down regulated in the *twl*∆ mutant. Overexpression of *TFB5* significantly suppressed the conidiation defects in the *twl*∆ mutant. Furthermore, Tfb5-GFP translocates to the nucleus during the phototropic response and under redox stress, while it failed to do so in the *twl*∆ mutant. Thus, we provide mechanistic insight into Twl-based regulation of nutrient and redox homeostasis in response to light during pathogen adaptation to the host milieu in the rice blast pathosystem.

## Author Summary

Growth in response to light, Phototropism, is important for differentiation and/or environmental adaptation in eukaryotes. Studies in plants and fungi revealed that light perception is tightly regulated by Clock genes, and displays circadian rhythm. On the other hand, carbon homeostasis and/or nutrient distribution have been shown to be under photoperiodic control, and thus robust growth and differentiation are optimized according to specific physiological state(s). Although a few photo-sensory proteins have been characterized in fungi, the exact downstream events triggered by light have not been explored in detail. Our study shows that a novel circadian clock-regulated gene, *TWL/Twilight*, is essential for proper asexual differentiation and pathogenicity in the rice-blast fungus *Magnaporthe oryzae*. Twilight coordinates (internal or *in planta*) carbon source utilization upon light perception. Transcriptional and posttranslational regulations enable Twilight to integrate and coordinate developmental (asexual reproduction), metabolic (carbon/nitrogen homeostasis), and environmental signals (light and reactive oxygen species) during the disease cycle in the rice blast pathogen. Lastly, we suggest that Twilight-based mechanisms allow the blast pathogen to entrain its metabolism in sync with optimal phototropic growth conditions in the host.

## Introduction

In eukaryotes, the phototropic response is important for growth and developmental adaptation to the environment [1,2,3,4,5]. Light perception is tightly regulated by the Clock genes, and displays circadian rhythmicity in plants [6,7]. In *Neurospora crassa*, a negative feedback loop, composed of *FRQ* (*FREQUENCY*, mRNA and protein) and WCC (White collar complex), drives robust, rhythmic sporulation at approximately 24-hour periodicity [8,9]. *M*. *oryzae* is an important fungal pathogen that causes the devastating Blast disease in rice and several crops [10]. *M*. *oryzae* initiates its pathogenic life cycle by forming asexual spores, conidia, upon exposure to light [11,12]. However, current knowledge on signaling and regulation of *M*. *oryzae* conidiation is limited. The *WC1* (*White Collar 1*) ortholog in *M*. *oryzae* has been found to be involved in sensing red light and in release of conidia [12]. However, conidia production was not affected in *M*. *oryzae wc1*∆ mutant [12]. Our recent studies showed that carbohydrate catabolism and homeostasis are spatially and temporally regulated via autophagy to ensure successful conidiation in *M*. *oryzae* [13,14]. The protein kinase Snf1 is involved in carbohydrate homeostasis by de-repression of genes repressed by the presence of glucose [15], and in induction of autophagy, in budding yeast [16]. The Snf1 ortholog in *M*. *oryzae* was shown to be essential for conidiation [17], but the substrates of MoSnf1 kinase have not been identified thus far.

Upon dispersal by wind, conidia get into contact with the host surface and start to differentiate infection-related structures called appressoria [11,18,19]. An appressorium is a dome-shaped cell at the tip of the conidial germ tube. Huge turgor is generated within the appressorium to facilitate physical breach of host cuticle and thus initiate invasive growth eventually leading to colonization of the host and multiplication of the fungal pathogen through next round of conidiation [20,21]. During *in planta*/invasive growth, *M*. *oryzae* encounters elevated ROS levels as part of the host resistance response [22], such that the ability to suppress such oxidative stress is critical for establishing pathogenesis.

Besides a role for the light sensor Wc1 [12], there are several observations that indicate that *M*. *oryzae* conidiation and its subsequent pathogenic development may be gated by circadian rhythm: 1) conidia formation requires light (day-time), while its release and vegetative growth requires dark (night-time) [12]; 2) host infection prefers dark, humid environment (night-time) [20]; 3) A close ortholog of the core circadian clock regulator, *Frequency* (*FRQ*), is present in *M*. *oryzae*, but has not been characterized thus far. Therefore, the circadian rhythm is likely important for asexual development and pathogenesis in *M*. *oryzae*. However, knowledge on the environmental inputs and the coordinated regulation of circadian rhythms in pathogenic fungi and their cognate hosts is limited and inadequate.

Here, we show that a novel protein named Twilight/Twl plays a key role in conidiation and pathogenesis in the blast fungus *M*. *oryzae*. GFP-Twl localizes predominantly to the nucleus upon photo-induction, but is largely present as cytosolic punctae during the dark phase. We show that unique post-translational modifications, de-acetylation and phosphorylation, drive such translocation of Twl into the nucleus in *M*. *oryzae*. Phosphorylation of Twl is catalyzed by the carbon sensor Snf1 kinase. A basal transcription factor Tfb5 is induced by Twl, and in turn switches on a set of downstream genes involved in carbon metabolism. We propose that Twl coordinates developmental (asexual reproduction and *in planta* growth), metabolic (carbon/nitrogen homeostasis), and environmental cues (light, ROS levels) in the blast fungus during its adaptation to and establishment within the host plants.

## Results

### Twilight is a circadian-regulated protein in *M*. *oryzae*

**Fig 1:**
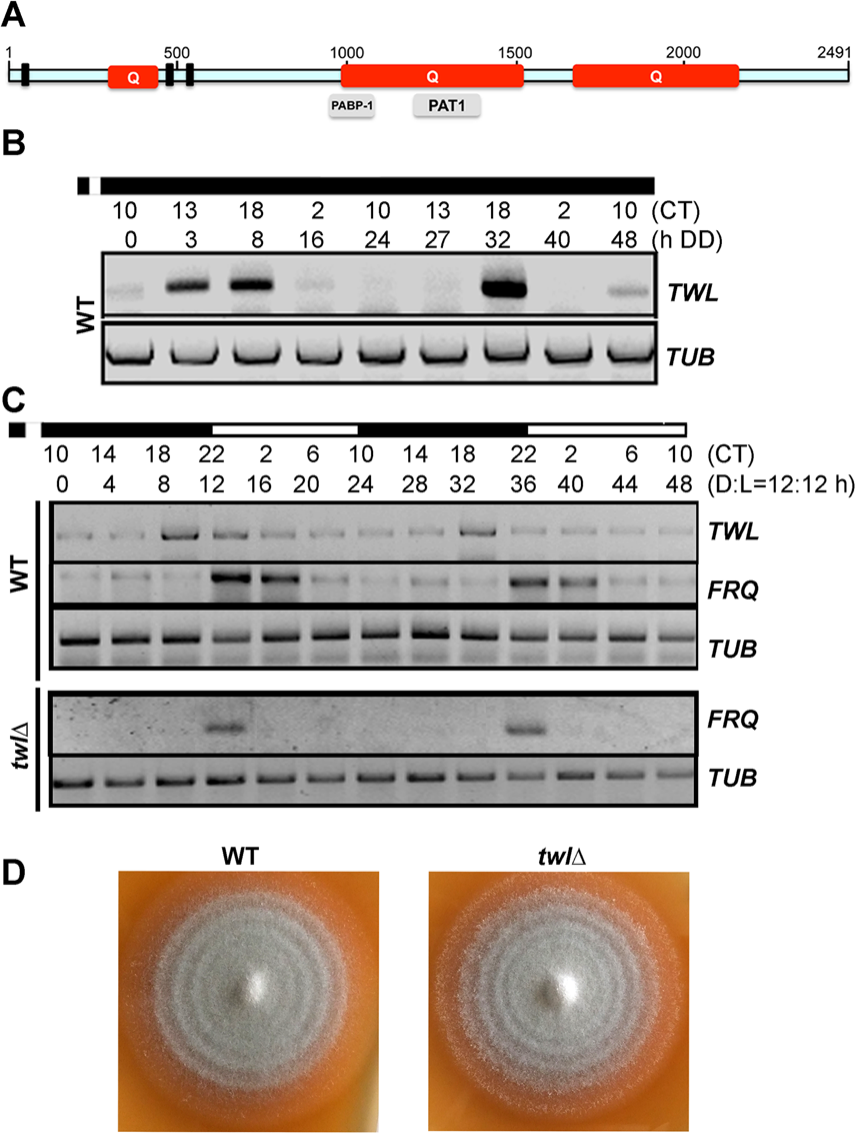
Twl is a novel circadian protein in *M*. *oryzae*. (A) Schematic representation of the domains in *M*. *oryzae* Twl protein sequence. Q, glutamine-rich region. Three short bars represent P-loop pattern [AG]-x(4)-[AG]-[KR]-[ST], identified by ScanProsite (http://prosite.expasy.org/scanprosite/). Grey bars in lower panel are partial domains predicted by NCBI. The schematic is drawn to scale based on the amino acid sequence. (B) Circadian rhythm of *TWL* in the wild type. WT mycelia were grown in the dark for 5 d, and then briefly (30 min) exposed to light, before a 48 h (two circadian cycles) growth in constant dark. Total RNA was extracted at the indicated time points for semi-quantitative reverse transcriptase PCR. CT, Circadian Time. CT12 corresponds to the time point immediately following light exposure. (C) Circadian rhythm of *TWL* and *FRQ* in the entrained wild type or the *twl*Δ mutant. WT or the *twl*Δ mycelia were grown in liquid Complete Medium (CM) in dark for 3 d, and then briefly (30 min) exposed to light, before a 48 h (two Dark/Light = 12h /12 h cycles) growth. Total RNA was extracted at four-hour intervals for semi-quantitative reverse transcriptase PCR. CT, Circadian Time. CT12 corresponds to the time point immediately following light exposure. (D) WT and the *twl*Δ mycelial plugs were inoculated on Prune Agar (PA) medium and kept in constant dark for three days, and then shifted to dark/light (12h /12 h) cycles. The conidial banding patterns in the WT and the *twl*Δ colonies were imaged after 4 dark/light cycles.

We identified *MGG_02916* in an RNA-Seq transcriptome analysis as a differentially expressed gene during photo-induced conidiation in the blast fungus *M*. *oryzae*. The expression of *MGG_02916* was five-fold higher (Light/Dark ratio = 5.02±0.31, p<0.001) in the light compared to dark, as shown by real-time RTPCR analysis. Based on cDNA sequence analyses, the deduced MGG_02916 protein was found to possess poly-Serine stretches containing potential phosphorylation site(s), a cluster of ATP/GTP binding site A (P-loop) pattern [AG]-x(4)-[AG]-[KR]-[ST] implicated in nuclear localization [23], and several Glutamine-rich regions (Fig 1A). Within the second Glutamine-rich region, there are two predicted domains with partial and weak similarity to PABP-1 (polyadenylate binding protein, human types 1, 2, 3, 4 family; TIGR01628) and PAT1 (Topoisomerase II-associated protein; pfam09770) motifs, spanning residues 956 to 1083, and 1194 to 1417, respectively (http://www.ncbi.nlm.nih.gov/protein/XP_003720804.1).

The *MGG_02916* transcript accumulated at 3h, 8h and 44h in constant dark following a short pulse of light (DD; Fig 1B). It is known that release of *M*. *oryzae* conidia occurs in the field at night, peaking between midnight and 7 am (before sunrise), and such conidia release is suppressed even by a short exposure to dim light [12]. Therefore, we defined the time point when *M*. *oryzae* resumes spore release as CT12 (circadian time 12, corresponding to 12 am/midnight), which makes the time point of light pulse given to be around CT10 (Fig 1B). We observed that in two continuous circadian cycles, the *MGG_02916* transcript peaked at CT18, corresponding to subjective dawn before sunrise (6 am). Furthermore, the rhythmic oscillation of the *MGG_02916* transcript was also observed in mycelia grown in liquid medium over two dark/light (12 h/12 h) cycles following a light pulse to synchronize the culture, and peaked at CT18 too (Fig 1C). Thus, we designated *MGG_02916* as *TWiLight* (*TWL*).

A *twl*Δ strain was generated by replacing the entire *MGG_02916* ORF with the hygromycin-resistance marker cassette (*HPH1*; S1A Fig), and confirmed by Southern blotting (S1B Fig). We assessed the rhythmic oscillation of the transcript of the conserved circadian clock regulator *FREQUENCY* (*FRQ*) in the wild type and *twl*∆ strain. *FRQ* accumulated at the start of the light phase, in the wild type as well as the *twl*∆ strain (Fig 1C). This result suggests that circadian rhythm may not depend on Twl function in *M*. *oryzae*. This was further supported by the observation that the mycelial/conidial banding pattern [12] under dark/light (12h /12 h) cycles was comparable between the wild type and the *twl*∆ mutant (Fig 1D). Therefore, we conclude that the *TWL* transcript displays circadian-regulated oscillations but is itself not a core component or regulator of the circadian clock in *M*. *oryzae*.

### Twilight function is necessary for proper conidiation and pathogenesis in *M*. *oryzae*

**Fig 2:**
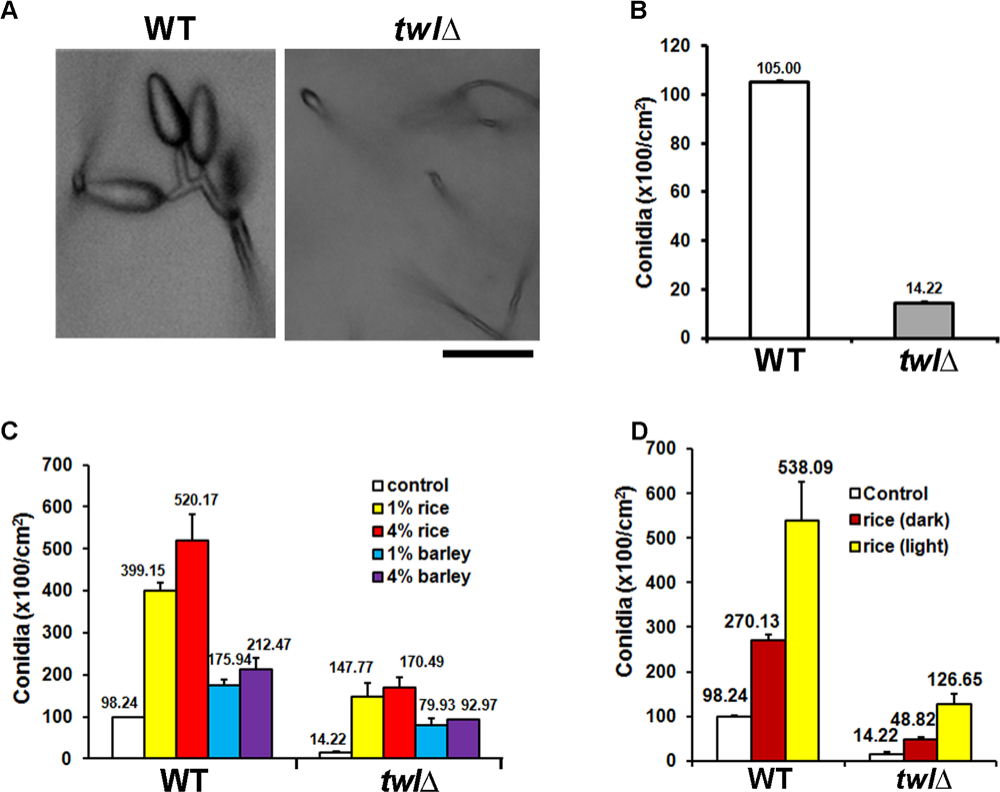
Twl is essential for proper conidiation in *M*. *oryzae*, likely by integrating nutrient homeostasis with phototropism. (A) Microscopic analyses of conidiating structures from wild type or *twl*Δ mutant. Images were taken 24 h post photo-induction. Scale bar = 10 micron. (B) Bar chart depicting quantitatively assessed conidiation in wild type and *twl*Δ. Mean values (±SE) presented as total number of conidia were derived from three independent experiments (n = 30 colonies for each sample). Assessments were performed 2 days post induction. (C) Bar chart depicting quantitative assessment of conidiation in wild type or the *twl*Δ supplemented with 1% or 4% rice or barley extract. (D) Bar chart depicting quantitative assessment of conidiation in wild type or the *twl*Δ supplemented with 1% rice extract from dark-grown (for 3 days) or light-grown CO39 seedlings.

Microscopic analysis and quantification revealed that asexual development (conidiation) was greatly reduced and aberrant in the *twl*∆ mutant (Fig 2A and 2B). Radial growth and colony morphology of the *twl*∆ were comparable to the wild type, when cultured under constant dark condition (S1C Fig). However, upon exposure to light to induce conidiation, the *twl*∆ colonies showed dark pigmentation and displayed significantly reduced aerial hyphal growth that imparted a flattened appearance (S1C Fig). Conidiophore formation from aerial hyphae was abundant in the wild type, while very rare in the *twl*∆ (S1D Fig). Conidiation was significantly improved in the *twl*∆ mutant in a dose-dependent manner with exogenous rice extract as the sole carbon source (Fig 2C). Interestingly, barley extract could promote conidiation in both wild type and the *twl*∆, but to a reduced extent and reached saturation at a comparatively lower concentration (Fig 2C). We further compared the nutrients derived from a compatible rice host (CO39) grown in constant dark or light phase, and found that the light-grown host could provide more favorable nutrients in promoting conidiation in wild-type *M*. *oryzae*, upon light induction (Fig 2D). The conidiation defect in the *twl*∆ mutant could be significantly suppressed by extracts from dark-grown host, although to a lesser extent compared to the light-grown host extract (Fig 2D). This result indicates that *M*. *oryzae* prefers nutrients derived from its native host, in a suitable growth phase (day-time, in the presence of light), while Twl may function in connecting these two important external stimuli, light and nutrients, to initiate conidiation.

**Fig 3:**
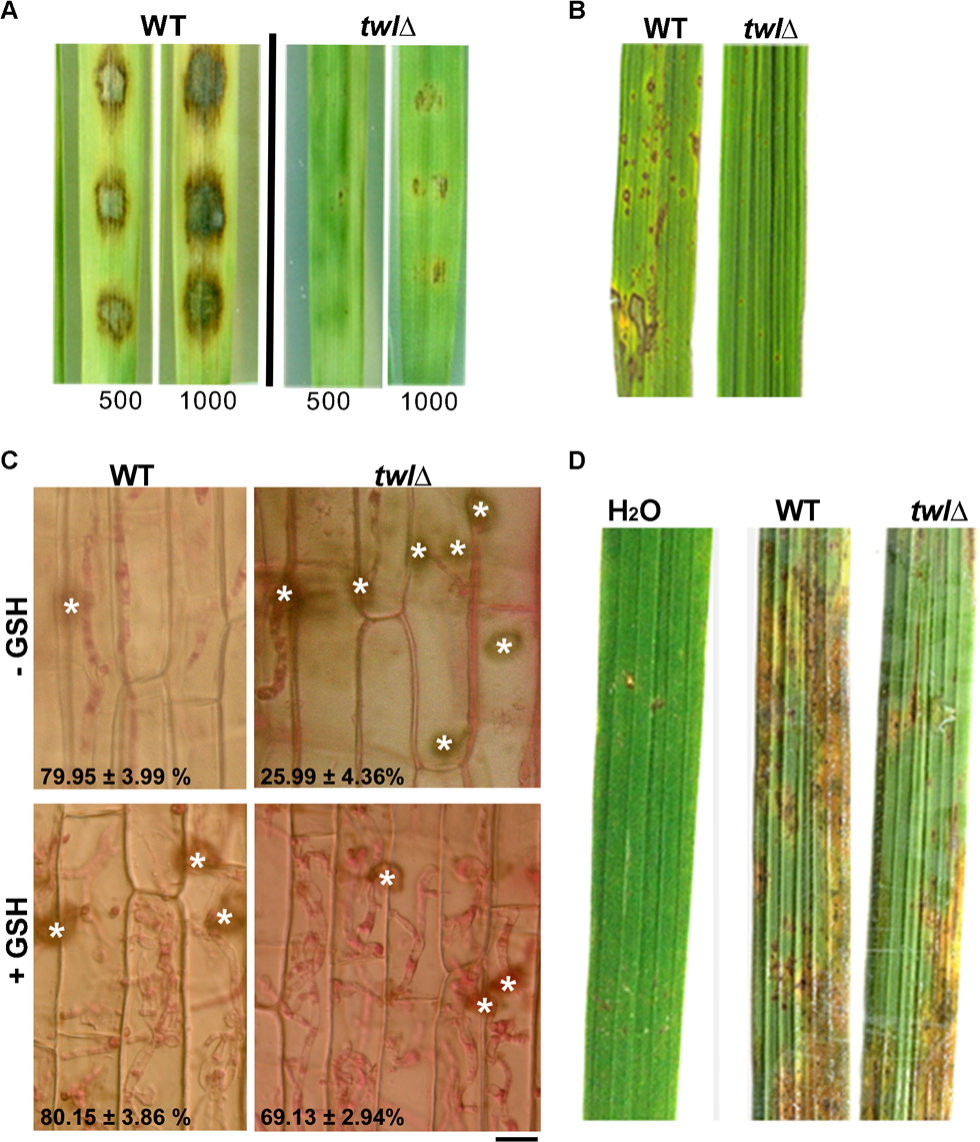
Twl facilitates redox homeostasis during *M*. *oryzae* pathogenicity. (A) Barley leaf explants were inoculated with conidia from the wild type or *twl*Δ. Disease symptoms were assessed after 5 days. Inoculum size (total number of conidia per droplet) is indicated accordingly. (B) Rice leaves from the susceptible cultivar IR31917 were inoculated with conidial droplets from wild type or *twl*Δ. Symptoms were examined at day 7 post inoculation. (C) Microscopic observation of *twl*Δ invasive hyphae developing in rice leaf sheath at 48 hpi, stained with 1% acid fuchsin. Mean values ± SE represent percentage of appressoria that differentiate invasive hyphae. Bar = 10 μm. For GSH treatment, L-Glutathione reduced (at a final concentration of 5 mM in water) was added to conidial droplets on rice leaf sheath at 24 hpi. (D) Rice leaves from 3 week-old IR31917 (blast susceptible) seedlings were wounded prior to inoculation with conidial suspension from wild type or *twl*Δ. The blast disease symptoms were evaluated 7 days post inoculation.

Barley and rice infection assays showed that the *twl*∆ mutant was significantly reduced in pathogenicity (Fig 3A and 3B). Microscopic observation with infected rice leaf sheath showed retarded development of *twl*∆ invasive hyphae, most (around 75%; *p* value < 0.05) of which lacked the ability to cross the host cell wall and spread from the primary infection site to the neighboring cells (48 hpi; Fig 3C). Pathogenic microbes elicit innate immunity or defense response in plants, which is manifested through rapid accumulation of reactive oxygen species (ROS) and cell death at the site of pathogen invasion [22]. We reasoned that the defective invasive growth of the *twl*∆ mutant might be due to its inability to suppress/tolerate ROS produced by the host. Exogenous addition of glutathione (GSH), an antioxidant, effectively facilitated invasive growth of *twl*∆ mutant (Fig 3C), suggesting that Twl is likely involved in regulating host-derived ROS levels *in planta*. Compared to the wild type, the *twl*∆ mutant showed increased sensitivity to oxidative stress exerted by hydrogen peroxide or menadione (Table 1). Furthermore, infection by *twl*∆ conidia was significantly improved upon wounding the rice leaf prior to inoculation (Fig 3D). Wounding of the host facilitates the access to host nutrients in *twl*∆, thus bypassing the requirement of counter-defense mechanism against host ROS and/or the host resistance response.

**Table 1:** *twl*∆ shows increased sensitivity to oxidative stress.

Conc.		Colony radius (cm)	
		WT	*twl*∆
	0	1.58 ± 0.06	1.63 ± 0.03
	2.5	1.64 ± 0.06	1.52 ± 0.06
**H**_**2**_ **O**_**2**_ **(mM)**	5	1.55 ± 0.12	1.31 ± 0.01
	10	1.14 ± 0.12	0.89 ± 0.01
	25	1.35 ± 0.04	1.32 ± 0.04
**Menadione (μM)**	50	1.18 ± 0.03	0.97 ± 0.03
	75	0.91 ± 0.01	0.59 ± 0.01

Overall, by characterizing conidiation and pathogenesis phenotypes in the *twl*∆ mutant, we showed that Twl might coordinate fungal pathogenic differentiation with environmental cues, including dark-light cycles and the corresponding nutritional/metabolic oscillations in the host. In addition, pathogenic defects in the *twl*∆ mutant could also be due to the disruption of redox equilibrium and/or a consequence of perturbed carbon homeostasis during invasive growth in the host plants.

### Snf1-dependent phosphorylation drives the nuclear localization of Twl in response to phototropic cues during conidiation

**Fig 4:**
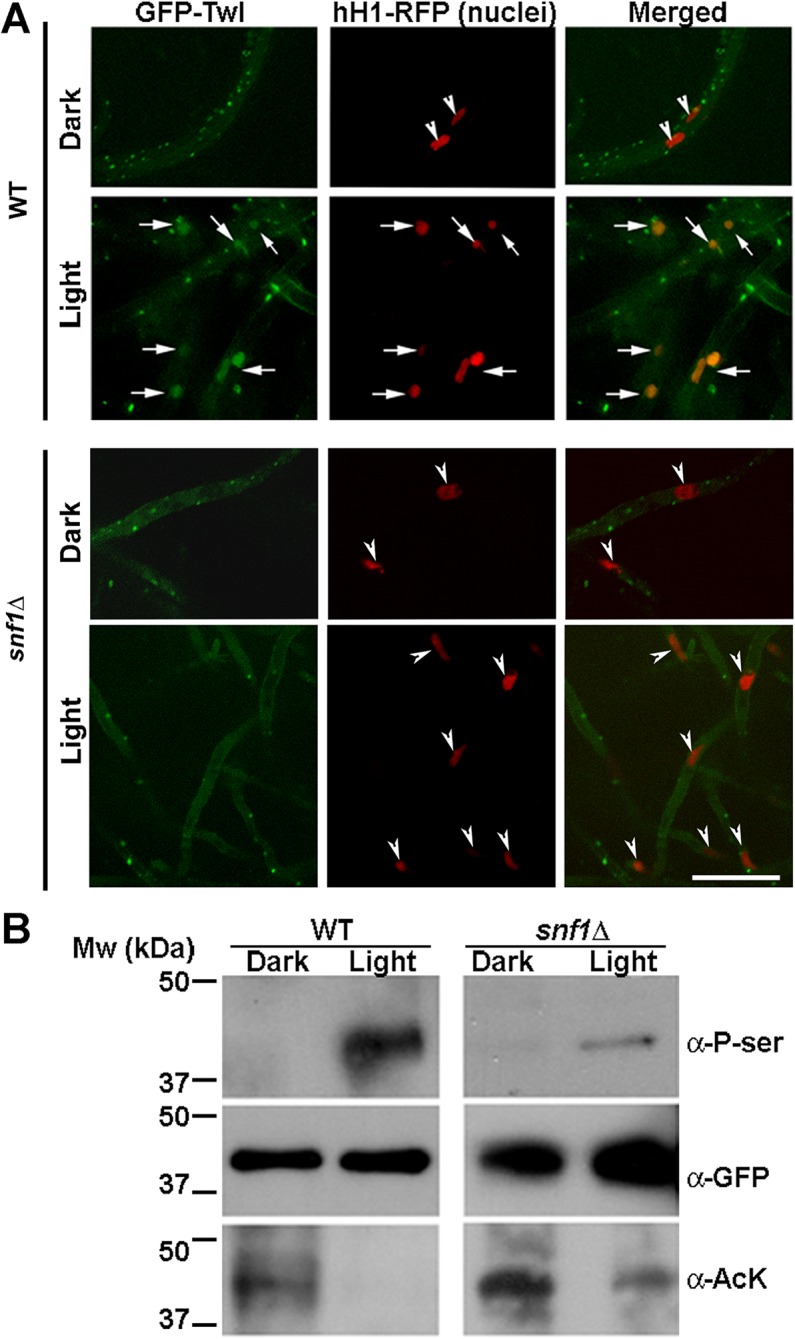
Snf1 kinase dependent phosphorylation drives the translocation of GFP-Twl into the nucleus in response to phototropic cues. (A) WT or *snf1*Δ mutant expressing GFP-Twl and hH1-RFP (as a marker of nuclei), were grown in constant dark or under constant illumination (12 h) and subjected to confocal microscopy. GFP-Twl co-localizes with nuclei marked with hH1-RFP in the WT in response to light exposure, but not in the *snf1*Δ mutant. Scale bar = 10μm. Arrows denote the overlap between GFP-Twl and the nuclei. Arrowheads denote nuclei (hH1-RFP) without GFP-Twl. (B) GFP-Twl is phosphorylated by Snf1 during phototropic growth. The GFP-Twl strain or *snf1*Δ expressing GFP-Twl was grown in the dark or subjected to photo-induction for 12 h before total protein extraction. GFP-Trap samples from the total lysates were probed with anti-PhoSer antibody to detect phosphorylation on Serine residue(s), and then stripped and re-probed with anti-GFP as a loading control. The same membrane was stripped and re-probed with anti-AcetLysine (anti-AcK) to detect possible acetylation on Twl.

Next, we investigated the subcellular localization of GFP-Twl by confocal microscopy. Numerous GFP-Twl punctae were evident in vegetative mycelia especially in the cultures exposed to light (Fig 4A). Co-localization of GFP-Twl and hH1-RFP, a nuclear marker (histone H1) tagged with RFP, was observed upon photo-induction during conidiation (Fig 4A, arrows in the lower panel for WT). In contrast, GFP-Twl was predominantly cytosolic in non-conidiating hyphae (grown under constant dark) and showed no overlap or association with nuclei (Fig 4A, arrowheads in the upper panel for WT). GFP-Twl punctae partially co-localized with the nuclear marker hH1-RFP in developing conidia, appressoria, and invasive hyphae (S2 Fig). We conclude that the phototropic response drives the translocation of GFP-Twl from the cytosol to the nucleus during conidiation and early stages of pathogenic development in rice blast.

The Snf1 kinase is known to be involved in de-repression of glucose-repressed genes [24], during carbohydrate metabolism under physiological conditions. *M*. *oryzae snf1*∆ has been shown to be defective in conidiation [17]. Given that conidiation is tightly regulated by carbon homeostasis [13,25], and that Twl contains potential phosphorylation sites and is likely involved in regulating nutrient homeostasis during dark/light cycles (Fig 2D), we assessed whether Snf1 phosphorylates Twl in response to light stimulus in *M*. *oryzae*. In contrast to the cytoplasmic/punctate (in dark) or nuclear (under light) localization in the wild type, GFP-Twl was predominantly cytosolic in the *snf1*∆ hH1-RFP mycelia grown in the dark or under constant light and failed to translocate to the nucleus (Fig 4A, arrowheads in *snf1*∆ panels).

Furthermore, we pulled down GFP-Twl from total lysates of dark- or light-grown *M*. *oryzae* cultures, and detected possible phosphoserine(s) peptides with the anti-P-Ser antibody. The phosphorylated form of GFP-Twl was specifically abundant in mycelial cultures exposed to light during conidiation, while undetectable or absent in the cultures grown in the dark (Fig 4B). We conclude that phosphorylation of Twl, likely via Snf1 kinase, leads to its localization in the nucleus, and is important for Twl function during phototropism in *M*. *oryzae*.

### Mutually exclusive acetylation and phosphorylation on Twl drives its nuclear translocation

Using mass spectrometry, we identified Sin3, a component of the Histone DeAcetylase Complex (HDAC), as one of the four proteins (Table 2) that physically interact with Twl-GFP. Recent studies revealed that HDAC (or its catalytic subunit/component) can also de-acetylate non-histone proteins [26,27]. To investigate whether Twl is acetylated, we assessed possible acetyl-lysine(s) with anti-AcK antisera in immuno-precipitated GFP-Twl. Interestingly, GFP-Twl was found to be acetylated in the dark-grown *M*. *oryzae* cultures, while such acetyl-lysine was undetectable in GFP-Twl from the mycelia cultures grown under light (Fig 4B). This raised at least two possibilities: 1) both phosphorylation and de-acetylation are required for nuclear localization of GFP-Twl; 2) only phosphorylation of GFP-Twl is essential for its transport into the nucleus, wherein it likely encounters HDAC. To differentiate between these two possibilities, we assessed the acetylation status of GFP-Twl in the *snf1*∆ mutant, and found that GFP-Twl remained acetylated even under the light (Fig 4B), which corresponds well with the lack of nuclear localization of acetylated GFP-Twl in this mutant background. Based on these results, we infer that a specific post-translational modification, acetylation, retains Twl predominantly in the cytosol during growth in the dark. We conclude that Snf1-based phosphorylation drives Twl into the nucleus and further propose that Twl is likely de-acetylated prior to or concomitant with the Snf1-catalyzed phosphorylation and the resultant transport into the nucleus during *M*. *oryzae* conidiation. It remains unclear whether GFP-Twl itself acts as a partner of HDAC for modification of histones, and thus exerts global transcriptional regulation.

**Table 2:** Specific protein interaction partners for Twilight identified using mass spectrometry.

Protein ID	Gene ID	Description
Swr1	*MGG_02183*	A component of Snf2/Swc3 complex for chromatin histone exchange
Sin3	*MGG_13498*	Forms histone deacetylase complex (HDAC) with Rpd3
Csn	*MGG_07656*	Chitosanase hydrolysing chitosan residues produced by partial or full deacetylation of chitin
Thnr	*MGG_02252*	Tetrahydroxynaphthalene reductase involved in melanin synthesis

### Endogenous cleavage of Twl releases an N-terminal short peptide that is sufficient for promoting *M*. *oryzae* conidiation

**Fig 5:**
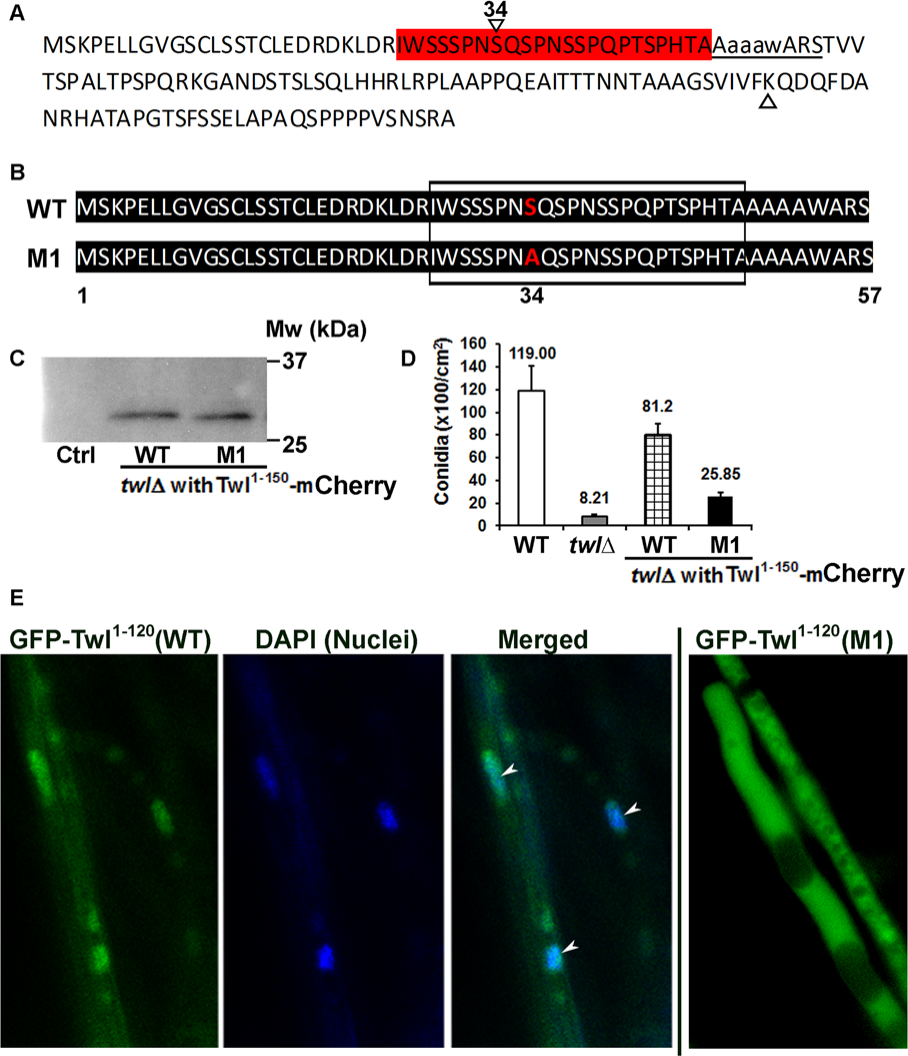
N-terminal phosphorylated Twl peptide is important for conidiation, but not pathogenicity of *M*. *oryzae*. **(A)** Predicted phosphorylated Serine residues (highlighted in red) and acetylated Lysine (marked with a triangle) in the N-terminal 1–150 aa peptide of Twl. Potential p-Ser site(s) are followed immediately by the first P-loop (underlined) in Twl. Serine 34 (denoted by reversed triangle and number 34 on top) was predicted as most likely phosphorylated site by Snf1. (B) Sequence alignment for the synthesized native (WT) or mutated (S34A; M1) 1–150 aa peptide of Twl. Boxed region depicts the predicted phosphor-Serine residues, corresponding to the region highlighted in red in (A) above. The serine at residue 34 that was mutated to alanine in the M1 variant peptide is highlighted in red. (C) Twl^1-150^-mCherry was pulled down with RFP-Trap and probed with anti-RFP antibody. Total protein from untagged wild type was included as a control (Ctrl). (D) Bar chart depicting quantitatively assessed conidiation in wild type, *twl*Δ and the *twl*Δ expressing either wild-type or the M1 Twl^1-150^ peptide). Mean values (±SE) presented as number of conidia per unit area were derived from three independent experiments (n = 30 colonies for each sample). Assessments were performed on day 2, post photo-induction. (E) The *twl*Δ expressing either wild-type or M1 GFP-Twl^1-120^ peptide was grown in constant dark for three days, before being exposed to light for 12 h. Arrowheads denote the GFP- Twl^1-120^ (WT) signal in the nuclei that were co-stained with DAPI. Scale bar = 5μm.

Interestingly, the size of the N-terminal phosphorylated Twl peptide fused with GFP that is pulled down by GFP-Trap is around 13 kDa, based on the relative mobility on western blots. This suggests that a shorter truncated version of Twl, likely the proximal 150 aa region, which contains the first P-loop, is sufficient for photo-induced phosphorylation and conidiation in *M*. *oryzae*. Bioinformatic analysis of this short peptide revealed a potential phosphorylation site (Fig 5A, highlighted red) and a Lysine (Fig 5A, green) residue for potential acetylation. We ectopically expressed mCherry-tagged native 150 aa or an S34A mutant derivative, termed M1, in the *twl*∆ strain (Fig 5B). Tagging with mCherry was meant to facilitate the subcellular localization of these two variants of Twl 1–150 aa peptides. However, a cleavage likely occurs between residues 120 and 150, releasing only a few amino acids tagged with mCherry (Fig 5C). Therefore, subcellular localization of Twl 1–150 aa (either WT or M1) could not be visualized via mCherry localization. However, this result confirmed that a natural endoproteolytic cleavage indeed occurs at the N-terminus of full-length GFP-Twl, giving rise to a GFP-tagged N-terminal Twl peptide (40 kDa, Fig 4B). We found that conidiation could be fully restored in *twl*∆ upon expression of the sequence encoding wild-type Twl^1-150^ (Fig 5D), while only marginally restored upon introduction of the S34A mutant Twl^1-150^ variant (Fig 5D). Neither WT nor the M1 Twl^1-150^ peptide was capable of restoring pathogenicity in the *twl*∆ mutant (S3 Fig). We managed to overexpress GFP-fused WT and M1 version of the N-terminal 1–120 aa (before the cleavage site) in the *twl*∆ strain. Subcellular localization of Twl^1-120^ (WT) was cytosolic in dark-cultured mycelia while it became nuclear upon light exposure (Fig 5E). In contrast, Twl^1-120^ (M1) remained cytosolic even under light condition (Fig 5E). Our results demonstrate that the phosphopeptide of Twl, derived from an N-terminal endoproteolytically cleaved fragment comprising of about 120 to 150 aa, is sufficient and essential for nuclear localization and photo-induced conidiation in *M*. *oryzae*.

### Twl facilitates induction of transcription/repair factor Tfb5

**Fig 6:**
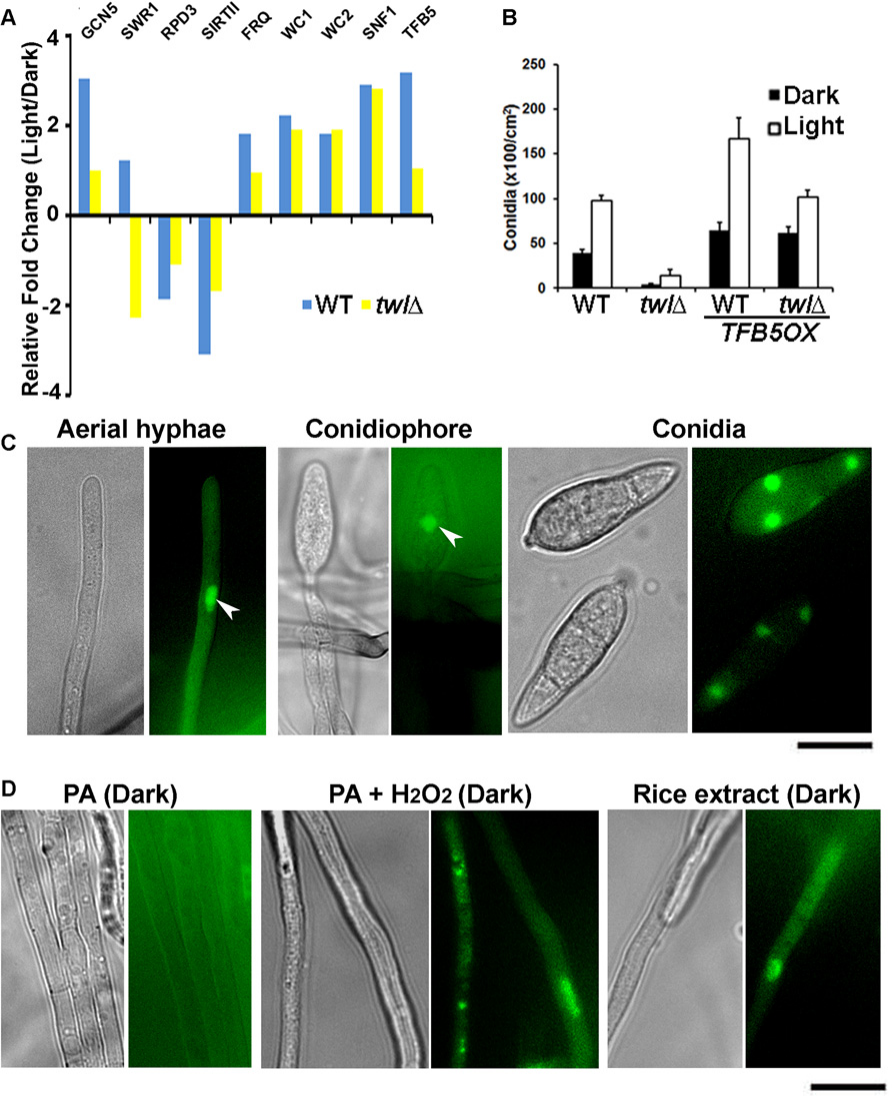
Transcriptional reprogramming during phototropic conidiation likely regulated by activation of transcription factor Tfb5. (A) Transcriptional regulation of genes encoding histone modifiers (*GCN5*, *SWR1*, *RPD3* and *SRT2*), circadian clock functions (*FRQ*, *WC1* and *WC2*), nutrient sensor (*SNF1*), or a transcription factor (*TFB5*), in response to photo induction. Wild type or *twl*Δ strain was grown in dark for 5 d, and exposed to light for 12 h. Dark or light grown mycelia were harvested for total RNA extraction and subjected to real time RTPCR analysis to detect the aforementioned transcripts. (B) *TFB5* overexpression suppresses the conidiation defects in *twl*Δ. Bar chart depicting quantitativeassessment of conidiation in wild type (WT) or *twl*Δ, with or without the overproduction of *TFB5*. (C) Tfb5-GFP localizes to nuclei of conidia, as well as in aerial hyphae and conidiophore under constant illumination. Scale bar = 5μm. (D) Nuclear Tfb5-GFP was induced by ROS or nutrients from host. Tfb5-GFP was cytosolic or undetected in dark grown mycelia on PA solid medium (left panel), while it became nuclear in mycelia grown on PA supplemented with rice extract (1%) or H_2_O_2_. Scale bar = 5μm.

By comparative transcriptomics, we identified several Differentially Expressed Genes (DEGs) during photo-induced conidiation in WT and the *twl*∆ mutant. Among such DEGs, we noticed that some of the histone modifiers, including Swr1 and Rpd3 (which physically associate with Twl; Table 2) and SirtII, (a histone deacetylase), are differentially regulated in the *twl*∆. Only one transcription factor, *TFB5*, showed differential regulation in the *twl*∆ mutant compared to the wild type during conidiation. *TFB5* may potentially act as a key regulator of *M*. *oryzae* conidiation and is likely subject to Twl-HDAC regulation. We carried out real time RTPCR to verify the differential expression of these selected transcripts between wild type and the *twl*∆ mutant, along with conserved circadian clock genes (*FRQ*, *WC1* and *WC2*) and *SNF1*. We found that *SWR1* was upregulated in response to light in the wild type, but down-regulated in the *twl*x (Fig 6A). Two histone deacetylases were transcriptionally down regulated in the wild type but were unchanged in *twl*∆ (Fig 6A). *FRQ* levels remained unperturbed upon loss of *TWL* (Fig 6A). A significant increase in the *WC1*, *WC2* and *SNF1* transcription was observed in response to photo-induction, but not dependent on Twl (Fig 6A). *TFB5* transcription is induced during conidiation while such induction is lost in the *twl*∆ (Fig 6A). Overall, we infer that Twl may not be involved in maintaining the free-running circadian rhythm but plays an essential role in light-responsive events, specifically, as a likely nuclear regulator that re-programs global gene expression (probably via HDAC-regulated gene transcription) during initiation of *M*. *oryzae* conidiation and pathogenesis.

As *TFB5* is transcriptionally induced during *M*. *oryzae* conidiation, we overexpressed it using *RP27* promoter, in the wild type or the *twl*∆ mutant. Conidiation was significantly higher in the resultant *TFB5OX* strain during dark as well as in photo-induced growth (Fig 6B). Interestingly, the conidiation defects in *twl*∆ could be fully suppressed upon *TFB5* overexpression (Fig 6B). In yeast, the *TFB5* gene is transcriptionally activated by the histone acetyltransferase Gcn5 [28], which is regulated by carbon homeostasis and oxidative stress conditions [29,30,31,32]. It has been shown that fungal Gcn5 plays a key role in conidiation [33,34], as well as in virulence [35]. Real time RTPCR analysis showed that *GCN5* was induced during the phototropic response in conidiation in *M*. *oryzae* as did *TFB5* (Fig 6A), indicating that Gcn5 may also act as a positive regulator of *M*. *oryzae* conidiation, likely via activation of *TFB5*.

A strain expressing *Tfb5*-*GFP* under native regulation was created to visualize the subcellular localization of Tfb5-GFP during *M*. *oryzae* conidiation. Nuclear Tfb5-GFP was observed in conidia, as well as in conidiation-related structures (Fig 6C; arrowheads for nuclear Tfb5-GFP in aerial hyphae and conidiophore). Interestingly, the overall percentage of aerial hyphae that showed Tfb5-GFP was 30.39 ± 7.71% (n = 120), falling into the range of overall conidiophore conversion out of the total aerial hyphae (20–40%, [13]). Therefore, we infer that Tfb5 potentially serves a conidiophore-specific function, differentiating conidiophore from other non-conidiating aerial hyphae. In contrast to photo-induced conidiating cultures, the dark-grown vegetative mycelia did not show any discernible Tfb5-GFP signal (Fig 6D, left panel). As Tfb5 is a potential target of Gcn5 regulation in response to ROS stress [32], we tested whether exogenous H_2_O_2_ was able to induce the nuclear Tfb5-GFP signal. Indeed, Tfb5-GFP was seen to localize in the nucleus in vegetative mycelia grown on PA medium supplemented with H_2_O_2_ (< 5 mM and not directly mixed into PA plugs with mycelia), even though the mycelia were kept in constant dark (Fig 6D, middle panel). Also, nutrients derived from light-grown compatible host were able to induce nuclear Tfb5-GFP in vegetative mycelia grown in constant dark (Fig 6D, right panel), suggesting that favorable nutritional source(s) could partially bypass the requirement of light for initiation of asexual development in *M*. *oryzae*, likely via inducing the levels of nuclear Tfb5 during conidiation.

**Fig 7:**
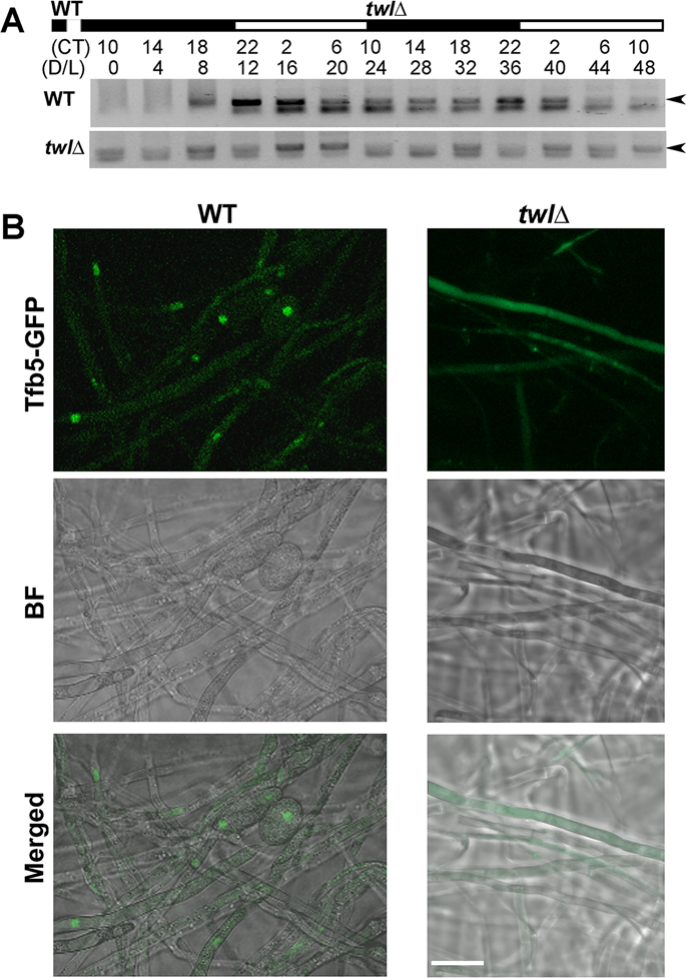
Twl regulates *M*. *oryzae* conidiation by rhythmic and phototropic induction of *TFB5*. (A) Circadian rhythm of *TFB5* in the entrained wild type or the *twl*Δ mutant. Sample processing, total RNA extraction and RT-PCR were performed as described for the experiments in Fig 1D. (B) Tfb5-GFP was nuclear in the WT, while weak or undetectable in the *twl*Δ mutant, at 12 h post photo-induction. Scale bar = 5μm.

Furthermore, we assessed Twl-dependent regulation of *TFB5* transcription during dark/light cycles, under the same conditions as mentioned in Fig 1C. We found that *TFB5* transcript accumulated in the light phase in the wild type (Fig 7A). In contrast, *TFB5* transcription was arrhythmic and overall at a lower level in the *twl*∆ mutant (Fig 7A). Lastly, Tfb5-GFP in the *twl*∆ mutant was indiscernible and was never present in the nucleus even upon photo-induction (Fig 7B). Therefore, we infer that Twl likely regulates *TFB5* expression and/or its nuclear localization, to induce *M*. *oryzae* conidiation in response to at least two external stimuli namely light and nutrient availability.

**Fig 8:**
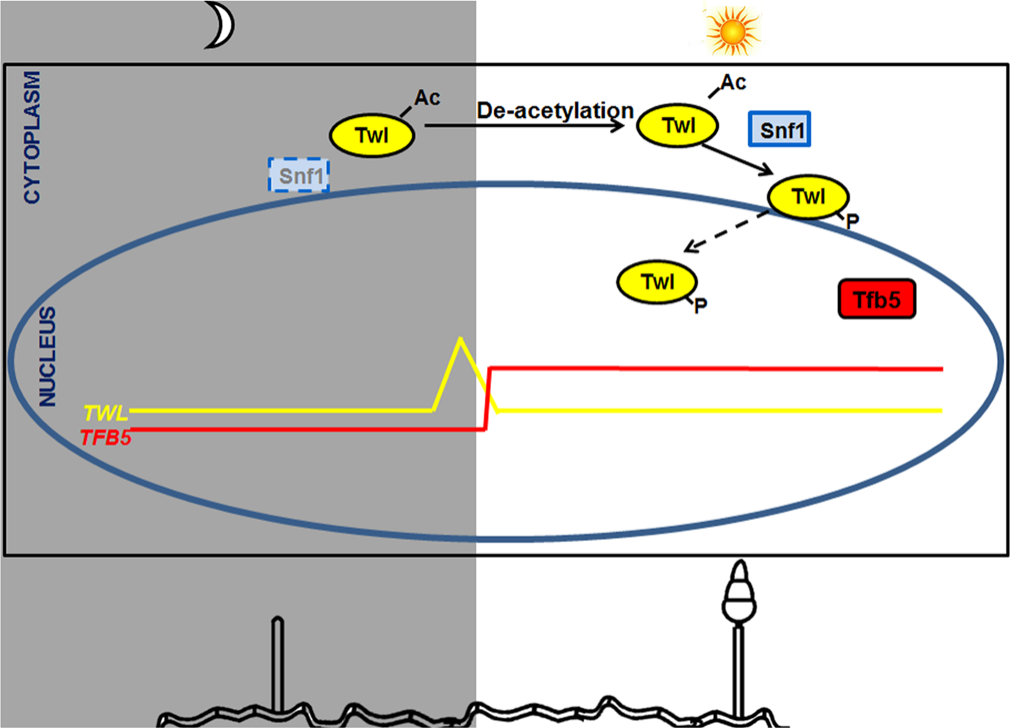
A proposed model for Twl-Snf1 regulated phototropic response during *M*. *oryzae* conidiation. *TWL* transcript peaks before sunrise and the Twl protein remains cytosolic and acetylated in the dark, while concomitant deacetylation and phosphorylation (by Snf1) of Twl occurs in response to light exposure. Phosphorylated Twl translocates into the nucleus and likely activates *TFB5*, which induces conidiation in *M*. *oryzae*. Snf1 is subjected to regulation by light (likely at the transcriptional level) and nutrient status (post-translational modification). Tfb5 transcription is likely regulated via Twl-mediated phototropic induction, and its nuclear localization directly influenced by host-derived nutrients.

Overall, we propose a model, that carbon sensing (Snf1) and phototropic induction of *M*. *oryzae* conidiation are linked or coupled by Twl likely as an output of the circadian cycle (Fig 8). Circadian accumulation of *TWL* transcript occurs at dawn, followed by phototropic modification and translocation of Twl protein into the nucleus. Consequently, conidiation is induced by activation of *TFB5* and its transcription targets (Fig 8). Nutritional input for inducing conidiation may act through regulation of Snf1 and/or Tfb5, and/or other as yet unidentified components in such phototropic response during initiation of blast disease in rice.

## Discussion

### Identification of a novel circadian-regulated gene function in phototropic conidiation of *M*. *oryzae*

*TWL* encodes a protein essential for proper asexual reproduction in *M*. *oyrzae*. The *TWL* transcript displays rhythmic oscillation during circadian cycles and accumulates before sunrise (twilight). Twl possesses three non-canonical nuclear localization consensus sequences, and three Glutamine-rich regions, within which it contains potential phosphorylation site(s). Nuclear localization of Twl is triggered by exposure to light, and is dependent on Snf1-catalyzed phosphorylation during asexual differentiation. A short span within the second Glutamine-rich region of Twl shows weak similarity to PABP-1 (polyadenylate binding protein, human types 1, 2, 3, 4 family) and PAT1 (Topoisomerase II-associated protein), respectively. Such molecular function of Twl is yet to be investigated.

In filamentous fungi, rhythmic conidiation occurs autonomously, yet is entrainable by light (phototropism), due to a negative feedback-defined circadian clock first identified in *N*. *crassa* [9]. Recent studies have added more circadian-regulated components that play important roles in fungal development, including (but not limited to) trehalose synthase encoded by *CCG9* (*Clock-Controlled Gene* 9) [36], Cellulose signalling associated protein Envoy [37], albino (al) genes involved in carotenoid biosynthesis [38], and superoxide dismutase Sod1 [39]. Our study adds a novel osciallating component, *Twilight*, that couples nutrient status and/or stress to phototropic conidiation. Another light and/or redox stress (ROS) induced transcript we identified in this study is *TFB5*, which could potentially serve as a biomarker for *M*. *oryzae* conidiation, and/or bio-control target for fungal pathogens.

### Cellular localization of Twilight in dark-light phase following unique posttranslational modification likely signaled by carbon/redox homeostasis

We observed GFP-Twl as cytoplasmic punctae in mycelia grown in the dark (Fig 4A). Co-staining with Mitoflour (mitochondrial marker) or Lysotracker (vacuoles), as well as co-localization with RFP-PTS1 (peroxisomes) ruled out the association between Twilight and the aforementioned organelles. GFP-Twl likely forms protein aggregates, as it possesses three Poly Q-rich regions, which have recently been shown to promote such aggregation in yeast [40].

Twilight likely plays pleotropic roles in *M*. *oryzae* development, as it translocates into the nuclei during phototropic conidiation (Fig 4A) as well as during early stages of pathogenicity (S2 Fig). Twl links nutrient (carbon) homeostasis with phototropic stimulus during conidiation, while regulating redox homeostasis during conidial development and/or *in planta* growth. However, these two pathways are likely inter-dependent and/or co-regulated and eventually mediated via downstream regulators such as the Tfb5 transcription factor.

GFP-Twl showed distinct subcellular localization between the dark and light phase, which is accompanied by posttranslational modification on Twl: The mRNA level of the transcription/repair factor TFB5, was significantly down regulated in Twl remains acetylated in the dark phase, correlating with its cytosolic localization; while de-acetylation and phosphorylation drives nuclear localization upon phototropic induction during conidiation. Such cytosol-nucleus shuttling was documented in the histone acetyltransferase Esa1 and the histone deacetylase Rpd3 for temporal regulation of autophagy via Atg3 in *S*. *cerevisiae* [41]. Atg3, thus serves as a non-canonical target of HAT or HDAC regulation [41]. GFP-Twl physically interacts with the Sin3/Rpd3 histone deacetylase complex and the chromatin modifier Swr1 (Table 2), indicating that Twl (Twilight) may be a potential, non-canonical substrate of HDAC. However, *M*. *oryzae* appears to lack the cytosol-nucleus shuttling of Rpd3 or Sin3 ortholog. The basal transcription factor Tfb5, a likely downstream target of Twl-HDAC regulated transcription, was also shown to translocate from the cytosol to the nucleus in response to environmental cues. The Tfb5 regulator, Gcn5, another histone acetyltransferase in yeast, was found to translocate into the nucleus in response to high level of oxygen [32]. Our study showed that the *GCN5* (*MGG_03677*) transcript increased in response to light exposure, likely in a Twl-dependent manner. It would be interesting to further investigate the subcellular localization of Gcn5 and the possible spatio-temporal regulation under different environmental and physiological conditions.

Another posttranslational modification on Twl, phosphorylation, was largely dependent on Snf1, a carbon sensor and kinase, indicating that carbon source metabolism may be linked to light sensing and response. GFP-Twl partially localized to nuclei in the invasive hyphae during pathogenic development, and such nuclear localization is likely in response to elevated oxidative stress, as a general host defense mechanism and/or a consequence of altered nutrient metabolism status linked to particular host milieu. Overall, we interpreted that Twl-Tfb5 signaling may respond to specific environmental clues, such as light and nutrients, to initiate pathogenic differentiation (conidiation or invasive growth) in *M*. *oryzae*.

### Metabolic and/or redox oscillation and phototropic conidiation

In nature, *M*. *oryzae* conidiation occurs following successful colonization of the host and depends on the nutrients derived *in planta*. Conidiation and infectious growth are interconnected and cyclical. Since Twl undergoes Snf1-catalyzed phosphorylation in response to light, we hypothesized that Twl likely integrates two important external stimuli: nutrients and light, during initiation of pathogenic differentiation. This hypothesis could be supported by the observation that *in planta* conidiation is poor in the *twl*Δ mutant, in contrast to the wild type (S4 Fig), presumably due to an inability to get access to and/or utilize the nutrients from the host. Differential utilization of various host nutrients may thus be mediated through the Snf1-Twl pathway. It is interesting that crude extracts from light-grown rice restored conidiation in the *twl*∆ to a higher extent than that from dark-grown rice. This indicates that accumulation of host nutrients may fluctuate/oscillate during the dark-light cycle, and that the requisite nutrient(s) that promote *M*. *oryzae* conidiation are likely more abundant during daytime, which coincides with such phototropic differentiation. Furthermore, host nutrients and ROS could induce*TFB5* expression and promote its nuclear localization, even in constant dark. Therefore, the time-of-day specific information from the host, including the metabolic and/or redox status, could be conveyed to the pathogen through induction of a hierarchy of signaling components (Snf1-Twilight-HDAC-Tfb5), and eventually entrain the pathogen growth and development in sync with the host. Our study on Twilight-based signaling sheds light on adaptive differentiation of pathogenic fungi, and potentially unravels a unique venue for developing antifungal strategies.

Lastly, the observed cyclical oscillation of *TWL* transcript and post-translational modification of Twl may be a consequence of co-evolution of pathogen with the host circadian rhythm, to ensure temporal entrainment of pathogen metabolism (and subsequent growth and propagation) that occurs in sync with the host milieu during establishment of the devastating blast disease in rice.

Future studies would investigate *TFB5* activation via acetylation-deacetylation cycles and screen for targets of the Twl/Tfb5 circuit, especially those involved in phototropism, fungal metabolism and host-pathogen interaction. Likewise, the upstream regulators of Twl function would be of interest together with the epigenetic modulators (if any) of the phototropic response in growth and development of the most important fungal pathogen of rice and several crop species.

## Materials and Methods

### Fungal strains and growth conditions

*M*. *oryzae* wild-type strain B157 (Field isolate, mat1-2) was obtained from the Directorate of Rice Research (Hyderabad). *M*. *oryzae* strains were propagated on prune-agar (PA) medium or complete medium (CM) as described [13]. Two-day old liquid CM-grown mycelia were ground in liquid nitrogen for the isolation of nucleic acids. To assess the growth and colony characteristics, *M*. *oryzae* isolates were cultivated on PA medium, at 28°C for a week. For total protein extraction from conidiating cultures, *M*. *oryzae* strains were either grown on PA in constant dark for 7 d, or allowed to grow under constant illumination for 2 d after 5 d of growth in the dark. Standard protocols were followed for quantitative analyses of conidiation, infection assays with barley leaf explants or rice leaf explants [13].

### Nucleic acid and protein-related manipulation

Fungal genomic DNA was extracted with the MasterPure Yeast DNA Purification Kit (Epicenter, MPY80200) following the manufacturer’s instructions. Plasmid extraction from E. coli was carried out using the Geneaid Mini Kit PD300. Recombinant DNA was examined by nucleotide sequencing using the ABI Prism Big Dye terminator method (PE-Applied Biosystems). Homology searches of DNA/protein sequences were performed using BLAST (http://blast.st-va.ncbi.nlm.nih.gov/Blast.cgi). Identification of domains in TicL was performed by SMART (http://smart.embl-heidelberg.de/) and recognition of [AG]-x(4)-[AG]-[KR]-[ST] pattern was done by ScanProsite (http://prosite.expasy.org/scanprosite/). The primers used for gene deletion, complementation and GFP tagging are listed in Table 3. Underlined text represents the restriction enzyme site introduced for cloning purpose. Reverse transcriptase PCR was performed using the one-step RT-PCR kit (QIAGEN, 210212). The primers for RT-PCR are summarized in Table 4. Quantitative real-time RTPCR was performed with ABI 7900HT Fast Real-Time PCR System, using KAPA SYBR FAST qPCR Kits (KK4605). The primers for real-time RTPCR were summarized in Table 4. Relative fold change was calculated with 2−∆∆CT method as reported [42]. Total protein extraction and immunoblotting analysis were carried out following previously described protocol. Primary antibodies used include: anti-GFP, Invitrogen- Molecular Probes, A6455; anti-RFP, Invitrogen- Molecular Probes, R10367; anti-Porin, Invitrogen, 459500; anti-PhoSer, Santa Cruz, sc-81514; anti-ACK, Acetyl Lysine, ab21623, at recommended dilutions. Secondary antibody conjugated to horseradish peroxidase was used at 1:20000. The Amersham ECL Kit (GE, RPN2135) was used to detect the chemiluminescent signals in immunoblotting experiments. Total RNA extraction from wild type and *twl*∆ was performed with Qiagen RNeasy plant mini kit (74904).

**Table 3:** Oligonucleotide primers used for plasmid construction in this study.

Gene (Locus)	Description	Enzyme sites	Primer sequence
*TWL* (*MGG__02916*.*6*)	Deletion construct	*-*	5’-GGGGCTCCTGGAATAGATTGGGGT-3’
		*-*	5’-ACCAGCAAGCAGGAAGCGGACGCG-3’
		*Pst*I	5’-GACTGTTCTGCAGGATGACATTGCATACAACGTCCAA-3’
		*Hind*III	5’-GAGTGTTAAGCTTATAGCGTCATGGTCTGGAATGAAC-3’
	GFP-tagging (N-	*Hind*III	5’-GAGTGTAAGCTTCCAGGTGGGTGCCAAGGGGAAGGAG-3’
	terminal) under RP27 promoter	*-*	5’-GACCCTTGTTGCATGAGGCCTGGG-3’
		*Kpn*I	5’- GAGAGTGGTACCATGTCCAAGCCTGAGCTTTTGGG -3’
		-	5’- CCGTAGCTCTGgaattcGACACAG-3’
		*Bam*HI	5’-GAGAGTGGATCCATAAATGTAGGTATTACCTGTA-3’
		*Nco*I	5’-GAGAGTGACCATGGTTTGAAGATTGGGTTCCTACGA-3’
		*Nco*I	5’-GAGAGTGACCATGGTGAGCAAGGGCGAGGAGCTGT-3’
		*Kpn*I	5’-GAGAGTGAGGTACCCTTGTACAGCTCGTCCATGCCGAG-3’
	genomic DNA for promoter and N-	*-*	5’-ggggctcctggaatagattggggt-3’
	terminal 150 aa genomic DNA for N-terminal 120 aa	*Xma*I	5’-GAGTGACCCGGGAGCTCTGGAATTCGACACAGGCGG-3’
		*Kpn*I	5’- GAGAGTGAGGTACCATGTCCAAGCCTGAGCTTTTGG-3’
		*-*	5’- GCCGCCTGTGTCGAATTCCAGA-3’
*SNF1* (*MGG_00803*.*6*)	Deletion construct	*Eco*RI	5’-GTGTGAATTCGGTGTGCCCAAAGACTGCCTTG-3’
		*Kpn*I	5’-GTGTGGTACCGGCGGATGGAGGGGATTGCTG-3’
		*Hind*III	5’-GTGTAAGCTTACCCAGGTCAATGCGATCGC-3’
		*Hind*III	5’-GTGTAAGCTTGCAACCGGAAGACTTGAGAG-3’
*TFB5* (*MGG_15089*.*6*)	GFP-tagging (N-terminal) under RP27 promoter	*Kpn*I	5’- GAGAGTGAGGTACCATGCCCAAGGCAGTCCATGGT -3’
		*Eco*RI	5’- GAGTGAGAATTCGCAAGAGCCCCGTGGCGGTGCAGG -3’

**Table 4:** Oligonucleotide primers used for reverse transcriptase PCR or real-time RTPCR in this study.

**Reverse transcriptase PCR**		
**Gene Name**	**Gene ID**	**Primer sequences**
*TWL*	*MGG_02916*.*6*	GGGAGACGCCGCCGGTAAT
		CGATTTGACCTCAAAGTTACCG
*FRQ*	*MGG_00903*.*6*	GCTATCGACCTCTCGGACACGGG
		ACGGAGCGTAGCCTCGAGCTCT
*TUB*	*MGG_00604*.*6*	AAACAACTGGGCCAAGGGTCACTACA
		CCGATGAAAGTCGACGACATCTTGAG
*TFB5*	*MGG_15089*.*6*	ATGCCCAAGGCAGTCCATG
		AGCCGGGCGGGCCTGCTTATCA
**Real time RTPCR**		
**Gene Name**	**Gene ID**	**Primer sequences**
*SWR1*	*MGG_02183*	TTATGCACCGGAAGTAGCTG
		GCCTTGACTAGGTGGTCCAT
*RPD3*	*MGG_05857*	ATGGGCATGACAGATGAAGA
		CCATTGCCTTTGTGTGATTC
*SIRII*	*MGG_10267*	GACGATTTAATGAGCGACGA
		CCGAATGATGAGCTGCTAAA
*FRQ*	*MGG_00903*.*6*	TCCACGAGTGTTCACTACAA
		GTGAACTTGAACCTTCTTCG
*WC1*	*MGG_03538*.*6*	TTGTCAAACACAGCTACTGG
		GTTGAGAAGTCGTTCGAGTC
*WC2*	*MGG_04521*.*6*	GCAACCGACAAGACTCTATC
		GGTCAACATCTCAATCGTCT
*TWL*	*MGG_02916*.*6*	TTTCTTCTCCAGATCTCCAA
		TCGTCGCAATTTAGTCTG
*SNF1*	*MGG_00803*.*6*	TTTCAAAGACCATGGGTTAC
		TCATGTACGCATCCTTGATA
*TFB5*	*MGG_15089*.*6*	CGATCAAGTCCATCATCGTC
		TCCTTGATCAACAAATGCGT
*GCN5*	*MGG_03677*.*6*	TTGCGCTATAAGCAGTGACC
		CCAATGGCATAATTGTCTGC
*TUB*	*MGG_00604*.*6*	GAGTCCAACATGAACGATCT
		GTACTCCTCTTCCTCCTCGT

### Generation of deletion mutants or epitope tagged strains

For gene deletion of *TWL*, genomic DNA fragments (about 1 kb each) representing the 5' and 3' UTR of *TWL* were amplified by PCR, ligated sequentially to flank the *HPH1* cassette. cDNAs were generated with AMV reversed transcriptase (Roche, 11495062001) and amplified by standard PCR. For RP27 Promoter-*GFP*-*TWL* constructs, the 1 Kb fragment from *TWL* promoter region and the first 500 bp of *TWL* coding sequence were amplified and ligated to frank PCR-amplified RP27 Promoter-*GFP* on the vector. By homologous recombination, RP27 promoter driven GFP was inserted in-frame at the N-terminus into the *TWL* locus, and thus constitutively express GFP-Twl. pFGL97 contains the *BAR* selection cassette. For *TWL* ^*1-150*^–*mCherry* constructs, genomic DNA sequence of the *TWL* promoter followed by the sequence encoding M1 variant of Twl^1-150^ peptide was custom synthesized (GenScript Inc. USA), while the genomic DNA for *TWL* Promoter with WT *TWL* ^1-150^ was PCR amplified with primers listed in Table 3. The amplified WT fragment or the synthesized M1 fragment was respectively inserted into the expression vector carrying mCherry coding sequence and *ILV1* cassette as selection marker, to produce (WT or M1) Twl^1-150^-mCherry fusion protein in transformed strains. For RP27 Promoter-*GFP*-*TWL* ^*1-120*^ constructs, genomic DNA sequence encoding either WT or M1 variants of Twl^1-120^ peptide was PCR amplified using the primers listed in Table 3, and with either the WT or synthesized M1 fragment as template. The amplified fragments were respectively inserted into the expression vector carrying *RP27* promoter-*GFP* construct and *BAR* selection marker. The resulting vector produced either WT or M1 GFP-Twl^1-120^ fusion protein in the *twlΔ* background. *hH1-RFP* was cloned into pFGL922 vector with homologous regions of native *ILV1* locus but introducing resistance to Sulfonylurea as a selection marker in the resultant transformants.

### Staining protocols and microscopy

To stain nuclei, conidiating cultures were incubated in 1μg/mL Hoechst (Invitrogen-Molecular Probes, 33258) solution for 15 min at room temperature, followed by a thorough wash with sterile water prior to confocal imaging. Epifluorescence was observed using a spinning disc confocal microscope (microLAMBDA Pte Ltd) or a Zeiss LSM510 inverted confocal microscope (Carl Zeiss Inc.) equipped with a 30 mW argon laser. The objective used was a 100 x Achromat (n.a. 1.25) oil immersion lens. EGFP was imaged with 488 nm wavelength laser excitation, using a 505 nm longpass emission filter, while RFP imaging with 543 nm laser excitation and a 560 nm long-pass emission filter.

## Supporting Information

S1 Fig Generation and characterization of *twl*Δ mutant. (TIF)

S2 Fig Subcellular localization of GFP-Twl during pathogenic development. (TIF)

S3 Fig Twl^1-150^ peptide is not sufficient for *M*. *oryzae* pathogenesis. (TIF)

S4 Fig *In planta* conidiation is compromised in the *twl*Δ mutant. (TIF)

## References

[1] Ruoff P, Vinsjevik M, Monnerjahn C, Rensing L (2001) The Goodwin model: simulating the effect of light pulses on the circadian sporulation rhythm of Neurospora crassa. J Theor Biol 209: 29–42. 11237568 10.1006/jtbi.2000.2239

[2] Hut RA, Beersma DG (2011) Evolution of time-keeping mechanisms: early emergence and adaptation to photoperiod. Philos Trans R Soc Lond B Biol Sci 366: 2141–2154. 10.1098/rstb.2010.0409 21690131 PMC3130368

[3] Henriques R, Jang IC, Chua NH (2009) Regulated proteolysis in light-related signaling pathways. Curr Opin Plant Biol 12: 49–56. 10.1016/j.pbi.2008.10.009 19084466

[4] Chaves I, Pokorny R, Byrdin M, Hoang N, Ritz T, et al (2011) The cryptochromes: blue light photoreceptors in plants and animals. Annu Rev Plant Biol 62: 335–364. 10.1146/annurev-arplant-042110-103759 21526969

[5] Cagampang FR, Bruce KD (2012) The role of the circadian clock system in nutrition and metabolism. Br J Nutr 108: 381–392. 10.1017/S0007114512002139 22676899

[6] Nakamichi N (2011) Molecular mechanisms underlying the Arabidopsis circadian clock. Plant Cell Physiol 52: 1709–1718. 10.1093/pcp/pcr118 21873329 PMC3189347

[7] Song YH, Ito S, Imaizumi T (2010) Similarities in the circadian clock and photoperiodism in plants. Curr Opin Plant Biol 13: 594–603. 10.1016/j.pbi.2010.05.004 20620097 PMC2965781

[8] Aronson BD, Johnson KA, Dunlap JC (1994) Circadian clock locus frequency: protein encoded by a single open reading frame defines period length and temperature compensation. Proc Natl Acad Sci U S A 91: 7683–7687. 8052643 10.1073/pnas.91.16.7683 PMC44466

[9] Crosthwaite SK, Loros JJ, Dunlap JC (1995) Light-induced resetting of a circadian clock is mediated by a rapid increase in frequency transcript. Cell 81: 1003–1012. 7600569 10.1016/s0092-8674(05)80005-4

[10] Ou SH (1985) Rice Diseases. Surrey, UK.

[11] Cole GT (1986) Models of cell differentiation in conidial fungi. Microbiol Rev 50: 95–132. 3523190 10.1128/mr.50.2.95-132.1986 PMC373060

[12] Lee K, Singh P, Chung WC, Ash J, Kim TS, et al (2006) Light regulation of asexual development in the rice blast fungus, Magnaporthe oryzae. Fungal Genet Biol 43: 694–706. 16765070 10.1016/j.fgb.2006.04.005

[13] Deng YZ, Ramos-Pamplona M, Naqvi NI (2009) Autophagy-assisted glycogen catabolism regulates asexual differentiation in Magnaporthe oryzae. Autophagy 5: 33–43. 19115483 10.4161/auto.5.1.7175

[14] Deng YZ, Naqvi NI (2010) A vacuolar glucoamylase, Sga1, participates in glycogen autophagy for proper asexual differentiation in Magnaporthe oryzae. Autophagy 6: 455–461. 10.4161/auto.6.4.11736 20383057

[15] Hedbacker K, Carlson M (2008) SNF1/AMPK pathways in yeast. Front Biosci 13: 2408–2420. 17981722 10.2741/2854 PMC2685184

[16] Wang Z, Wilson WA, Fujino MA, Roach PJ (2001) Antagonistic controls of autophagy and glycogen accumulation by Snf1p, the yeast homolog of AMP-activated protein kinase, and the cyclin-dependent kinase Pho85p. Mol Cell Biol 21: 5742–5752. 11486014 10.1128/MCB.21.17.5742-5752.2001 PMC87294

[17] Yi M, Park JH, Ahn JH, Lee YH (2008) MoSNF1 regulates sporulation and pathogenicity in the rice blast fungus Magnaporthe oryzae. Fungal Genet Biol 45: 1172–1181. 10.1016/j.fgb.2008.05.003 18595748

[18] Lee YH, Dean RA (1993) cAMP Regulates Infection Structure Formation in the Plant Pathogenic Fungus Magnaporthe grisea. Plant Cell 5: 693–700. 12271080 10.1105/tpc.5.6.693 PMC160306

[19] Gilbert RD, Johnson AM, Dean RA (1996) Chemical signals responsible for appressorium formation in the rice blast fungus Magnaporthe grisea. Physiol Mol Plant Pathol 48: 335–346.

[20] Hamer JE, Talbot NJ (1998) Infection-related development in the rice blast fungus Magnaporthe grisea. Curr Opin Microbiol 1: 693–697. 10066544 10.1016/s1369-5274(98)80117-3

[21] Talbot NJ (2003) On the trail of a cereal killer: Exploring the biology of Magnaporthe grisea. Annu Rev Microbiol 57: 177–202. 14527276 10.1146/annurev.micro.57.030502.090957

[22] Apostol I, Heinstein PF, Low PS (1989) Rapid Stimulation of an Oxidative Burst during Elicitation of Cultured Plant Cells: Role in Defense and Signal Transduction. Plant Physiol 90: 109–116. 16666719 10.1104/pp.90.1.109 PMC1061684

[23] Ding Z, Millar AJ, Davis AM, Davis SJ (2007) TIME FOR COFFEE encodes a nuclear regulator in the Arabidopsis thaliana circadian clock. Plant Cell 19: 1522–1536. 17496120 10.1105/tpc.106.047241 PMC1913727

[24] Celenza JL, Carlson M (1986) A yeast gene that is essential for release from glucose repression encodes a protein kinase. Science 233: 1175–1180. 3526554 10.1126/science.3526554

[25] Wilson RA, Jenkinson JM, Gibson RP, Littlechild JA, Wang ZY, et al (2007) Tps1 regulates the pentose phosphate pathway, nitrogen metabolism and fungal virulence. EMBO J 26: 3673–3685. 17641690 10.1038/sj.emboj.7601795 PMC1949003

[26] Yi C, Ma M, Ran L, Zheng J, Tong J, et al (2012) Function and molecular mechanism of acetylation in autophagy regulation. Science 336: 474–477. 10.1126/science.1216990 22539722

[27] Shubassi G, Robert T, Vanoli F, Minucci S, Foiani M (2012) Acetylation: a novel link between double-strand break repair and autophagy. Cancer Res 72: 1332–1335. 10.1158/0008-5472.CAN-11-3172 22422989

[28] Venters BJ, Wachi S, Mavrich TN, Andersen BE, Jena P, et al (2011) A comprehensive genomic binding map of gene and chromatin regulatory proteins in Saccharomyces. Mol Cell 41: 480–492. 10.1016/j.molcel.2011.01.015 21329885 PMC3057419

[29] Abate G, Bastonini E, Braun KA, Verdone L, Young ET, et al (2012) Snf1/AMPK regulates Gcn5 occupancy, H3 acetylation and chromatin remodelling at S. cerevisiae ADY2 promoter. Biochim Biophys Acta 1819: 419–427. 10.1016/j.bbagrm.2012.01.009 22306658 PMC3319277

[30] Friis RM, Wu BP, Reinke SN, Hockman DJ, Sykes BD, et al (2009) A glycolytic burst drives glucose induction of global histone acetylation by picNuA4 and SAGA. Nucleic Acids Res 37: 3969–3980. 10.1093/nar/gkp270 19406923 PMC2709565

[31] Avendano A, Riego L, DeLuna A, Aranda C, Romero G, et al (2005) Swi/SNF-GCN5-dependent chromatin remodelling determines induced expression of GDH3, one of the paralogous genes responsible for ammonium assimilation and glutamate biosynthesis in Saccharomyces cerevisiae. Mol Microbiol 57: 291–305. 15948967 10.1111/j.1365-2958.2005.04689.x

[32] Hickman MJ, Spatt D, Winston F (2011) The Hog1 mitogen-activated protein kinase mediates a hypoxic response in Saccharomyces cerevisiae. Genetics 188: 325–338. 10.1534/genetics.111.128322 21467572 PMC3122313

[33] Xin Q, Gong Y, Lv X, Chen G, Liu W (2013) Trichoderma reesei histone acetyltransferase Gcn5 regulates fungal growth, conidiation, and cellulase gene expression. Curr Microbiol 67: 580–589. 10.1007/s00284-013-0396-4 23748966

[34] Canovas D, Marcos AT, Gacek A, Ramos MS, Gutierrez G, et al. (2014) The Histone Acetyltransferase GcnE (GCN5) Plays a Central Role in the Regulation of Aspergillus Asexual Development. Genetics. 10.1534/genetics.114.165688 PMC4125392 24907261

[35] O'Meara TR, Hay C, Price MS, Giles S, Alspaugh JA (2010) Cryptococcus neoformans histone acetyltransferase Gcn5 regulates fungal adaptation to the host. Eukaryot Cell 9: 1193–1202. 10.1128/EC.00098-10 20581290 PMC2918934

[36] Shinohara ML, Correa A, Bell-Pedersen D, Dunlap JC, Loros JJ (2002) Neurospora clock-controlled gene 9 (ccg-9) encodes trehalose synthase: circadian regulation of stress responses and development. Eukaryot Cell 1: 33–43. 12455969 10.1128/EC.1.1.33-43.2002 PMC118043

[37] Tisch D, Kubicek CP, Schmoll M (2011) New insights into the mechanism of light modulated signaling by heterotrimeric G-proteins: ENVOY acts on gna1 and gna3 and adjusts cAMP levels in Trichoderma reesei (Hypocrea jecorina). Fungal Genet Biol 48: 631–640. 10.1016/j.fgb.2010.12.009 21220037 PMC3082050

[38] Li C, Sachs MS, Schmidhauser TJ (1997) Developmental and Photoregulation of Three Neurospora crassa Carotenogenic Genes during Conidiation Induced by Desiccation. Fungal Genet Biol 21: 101–108. 9073484

[39] Yoshida Y, Maeda T, Lee B, Hasunuma K (2008) Conidiation rhythm and light entrainment in superoxide dismutase mutant in Neurospora crassa. Mol Genet Genomics 279: 193–202. 18084778 10.1007/s00438-007-0308-z

[40] Lu K, Psakhye I, Jentsch S (2014) Autophagic clearance of polyQ proteins mediated by ubiquitin-Atg8 adaptors of the conserved CUET protein family. Cell 158 (3): 549–563. 10.1016/j.cell.2014.05.048 25042851

[41] Yi C, Ma M, Ran L, Zheng J, Tong J, et al (2012) Function and molecular mechanism of acetylation in autophagy regulation. Science 336: 474–477. 10.1126/science.1216990 22539722

[42] Livak KJ, Schmittgen TD (2001) Analysis of relative gene expression data using real-time quantitative PCR and the 2(-Delta Delta C(T)) Method. Methods 25: 402–408. 11846609 10.1006/meth.2001.1262

