# Supplementary figures and images for: Twilight, a Novel Circadian-Regulated Gene, Integrates Phototropism with Nutrient and Redox Homeostasis during Fungal Development
Source: PLoS Pathog. 2015 Jun 23;11(6):e1004972. doi: 10.1371/journal.ppat.1004972 (PMC4478003; doi:10.1371/journal.ppat.1004972)

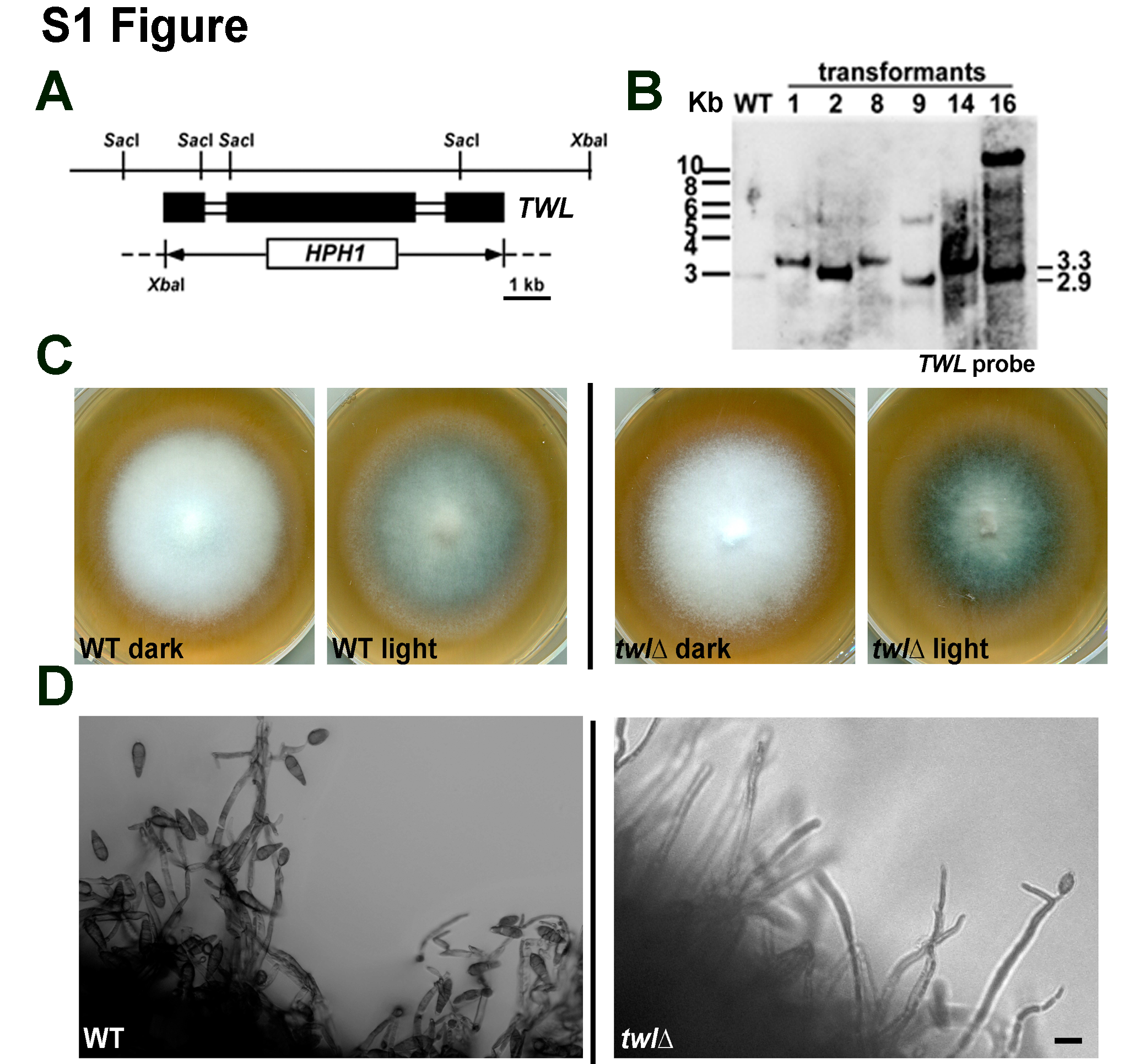

Supplement: S1 Fig — (TIF) [file ppat.1004972.s001.tif]

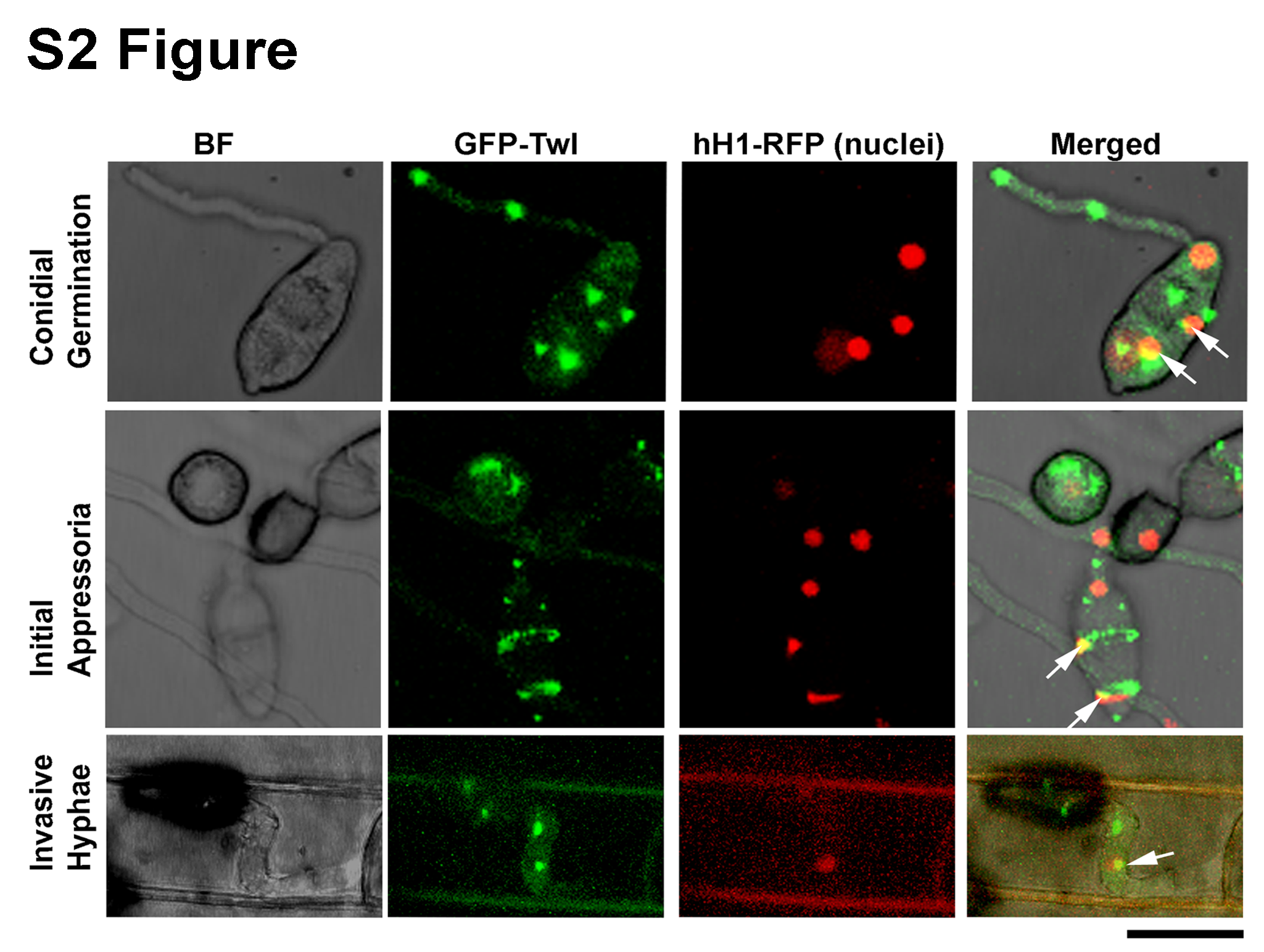

Supplement: S2 Fig — (TIF) [file ppat.1004972.s002.tif]

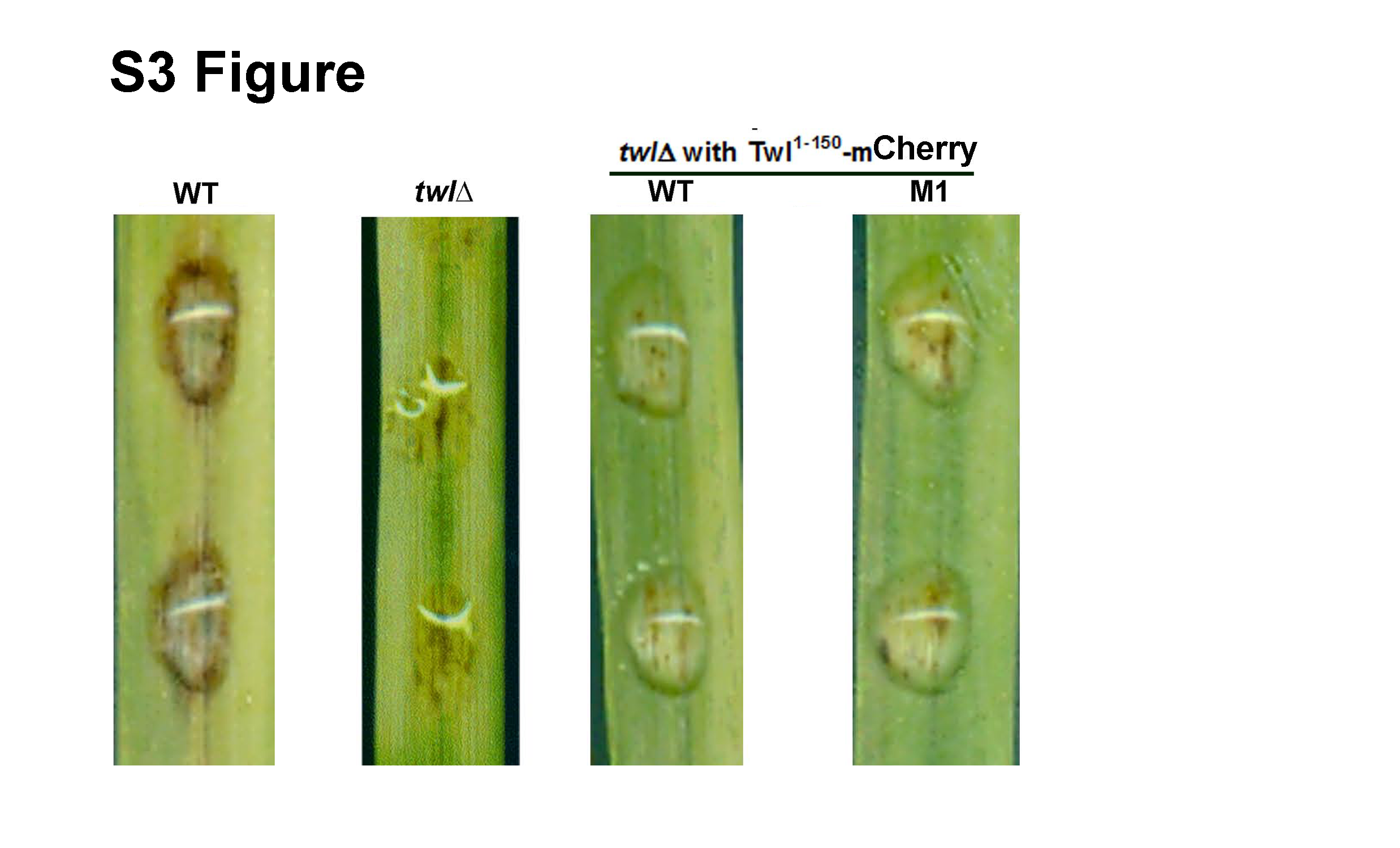

Supplement: S3 Fig — (TIF) [file ppat.1004972.s003.tif]

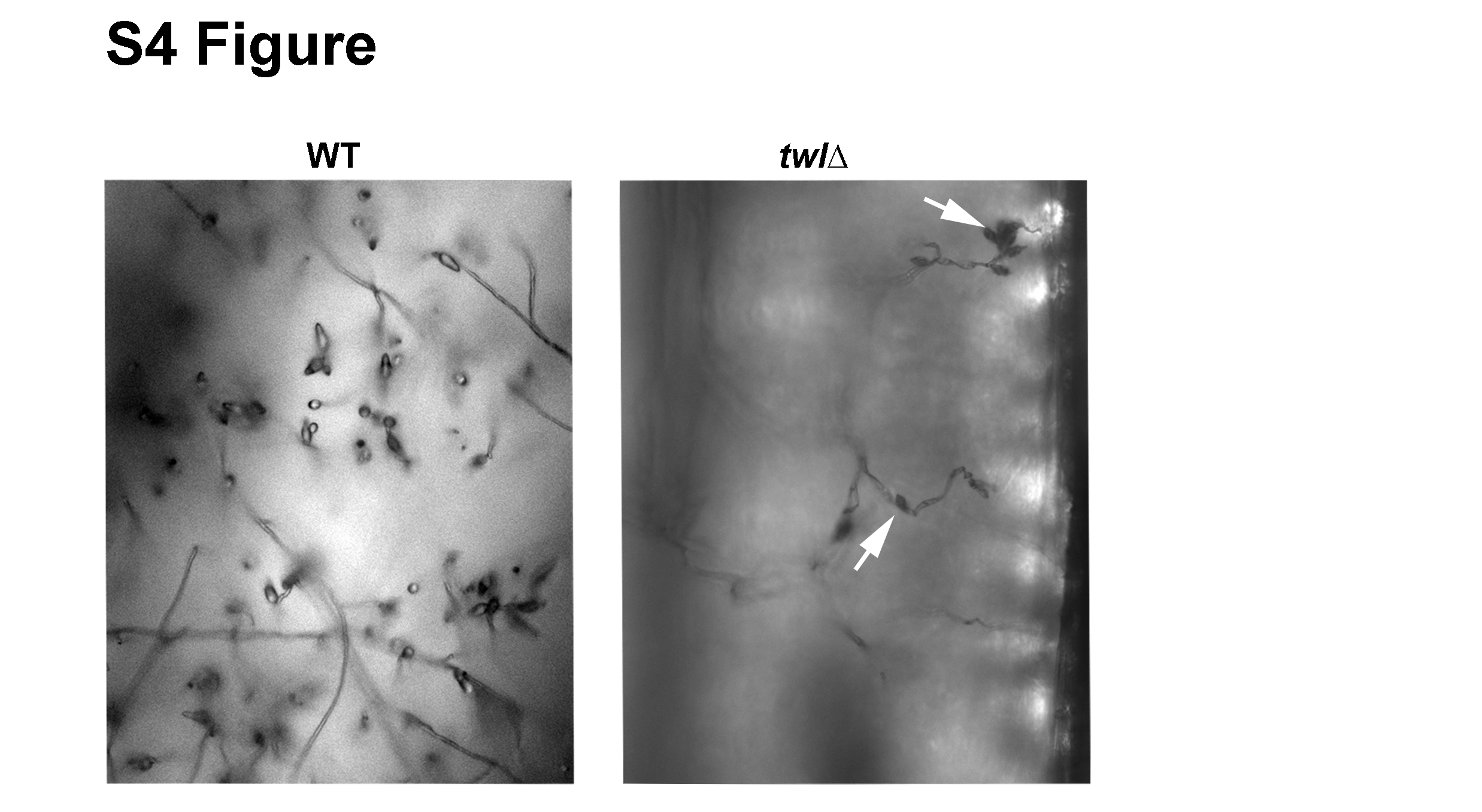

Supplement: S4 Fig — (TIF) [file ppat.1004972.s004.tif]
